# Effects of Four Formulations of Prostaglandin Analogs on Eye Surface Cells. A Comparative Study

**DOI:** 10.1371/journal.pone.0129419

**Published:** 2015-06-12

**Authors:** Fernando Pérez-Roca, Esther Rodrigo-Morales, Ingrid Garzón, Ana-Celeste Oliveira, Miguel-Ángel Martín-Piedra, Víctor Carriel, Ana-Isabel Ortiz-Pérez, Indalecio Sánchez-Montesinos, Antonio Campos, Miguel Alaminos

**Affiliations:** 1 Hospital General Básico de Baza, E18800, Granada, Spain; 2 PhD Program in Biomedicine, University of Granada, 18012, Granada, Spain; 3 Hospital La Inmaculada, Huércal Overa, 04600, Almería, Spain; 4 Tissue Engineering Group, Department of Histology, University of Granada, 18012, Granada, Spain; 5 Instituto de Investigación Biosanitaria ibs.GRANADA, 18012, Granada, Spain; 6 Department of Human Anatomy and Embryology, University of Granada, 18012, Granada, Spain; University of Reading, UNITED KINGDOM

## Abstract

We evaluated the cytotoxic effects of four prostaglandin analogs (PGAs) used to treat glaucoma. First we established primary cultures of conjunctival stromal cells from healthy donors. Then cell cultures were incubated with different concentrations (0, 0.1, 1, 5, 25, 50 and 100%) of commercial formulations of bimatoprost, tafluprost, travoprost and latanoprost for increasing periods (5 and 30 min, 1 h, 6 h and 24 h) and cell survival was assessed with three different methods: WST-1, MTT and calcein/AM-ethidium homodimer-1 assays. Our results showed that all PGAs were associated with a certain level of cell damage, which correlated significantly with the concentration of PGA used, and to a lesser extent with culture time. Tafluprost tended to be less toxic than bimatoprost, travoprost and latanoprost after all culture periods. The results for WST-1, MTT and calcein/AM-ethidium homodimer-1 correlated closely. When the average lethal dose 50 was calculated, we found that the most cytotoxic drug was latanoprost, whereas tafluprost was the most sparing of the ocular surface *in vitro*. These results indicate the need to design novel PGAs with high effectiveness but free from the cytotoxic effects that we found, or at least to obtain drugs that are functional at low dosages. The fact that the commercial formulation of tafluprost used in this work was preservative-free may support the current tendency to eliminate preservatives from eye drops for clinical use.

## Introduction

Glaucoma is the second most frequent cause of blindness worldwide, with over 11 million cases attributed to this disease [1,2]. Progression can be slowed by reducing intraocular pressure (IOP) pharmacologically [3,4]. Some of the most popular and efficient drugs for the treatment of glaucoma are prostaglandin analogs (PGAs). These drugs are currently considered the first line of treatment for glaucoma because of their efficacy and safety [5]. PGAs act primarily by enhancing uveoscleral outflow of the aqueous humor, and thereby decreasing IOP [6–8]. Four different PGAs are currently available in Europe for clinical use: travoprost, latanoprost, bimatoprost and tafluprost. However, the effect of these drugs on human cells are not well known.

In most cases chronic treatment with one or several drugs is necessary for the efficient control of IOP. Although most treatments are free from severe systemic effects, the chronic use of these drugs may be associated with local side effects in the long term. In this regard, several studies reported that the use of PGAs may lead to conjunctival hyperemia [9–11], increased iris pigmentation [12–14], changes in the biomechanical properties of the cornea [15], eye surface inflammation, dry eye syndrome and failure of filtration surgery [16–18]. However, the direct cytotoxic effects of PGAs for clinical use have not been fully established.

A number of studies have determined the cytotoxic effects of several PGAs and other ophthalmic agents. Most of these studies were done with animal cells [19,20] or immortalized human cell lines [21–24], and very few studies used normal human cells isolated from the conjunctiva [25,26]. In addition, very few studies have compared all four PGAs currently available in Europe [27]. Therefore, we lack sufficient knowledge about how the various PGAs may affect the ocular surface.

In this study we evaluated the cytotoxic effects of four PGAs on normal human conjunctival cell cultures at different concentrations and times, using three methods to determine the biosafety levels of the drugs used most commonly to control IOP.

## Materials and Methods

### Conjunctival stromal cell cultures

Primary cultures of human conjunctival stromal cells were established from small surgical biopsies obtained from 3 healthy donors undergoing eye surgery (i.e. strabismus or retinal detachment surgery) who had not been previously treated with eye drops. Average age of the donors was 50 years. Cells were isolated from tissue biopsies using a 2% (w/v) solution of type I collagenase from *Clostridium hystoliticum* (Gibco BRL Life Technologies Ref. 17100–017, Karlsruhe, Germany), corresponding to approximately 100 U/ml. Briefly, biopsies were incubated in the collagenase solution at 37C for 4–6 h until complete dissociation with slight agitation. Detached cells were then harvested by centrifugation, supernatant was discarded and the cell pellet was resuspended in DMEM with 10% fetal calf serum as culture medium (Sigma-Aldrich ref. D5796, Saint-Quentin-Fallavier, France) and transferred to culture flasks. Cells were cultivated at 37C in a 5% CO_2_ atmosphere using the same culture medium, and subconfluent cells were subcultured to new culture flasks using trypsin-EDTA solution (Sigma-Aldrich ref. T3924) until passage 6. All experiments were carried out on subconfluent cells corresponding to cell passages 3 to 6. All donors gave their written consent to participate in the study, and this study was approved by the San Cecilio University Hospital research and ethics committee.

### Study groups

Commercially available formulations of the four PGAs that are currently approved for clinical use were assayed in this work: 0.03% bimatoprost with 0.005% benzalkonium chloride (BAK) (Lumigan, Allergan, Irvine, CA), 0.005% latanoprost with 0.02% BAK (Xalatan, Pfizer, New York, NY); 0.0015% tafluprost without preservatives (Saflutan, Merck, Kenilworth, NJ), and 0.015% travoprost with 0.04% BAK (Travatan, Alcon Laboratories, Fort Worth, TX). For each product we studied 6 different concentrations of the commercial formulation diluted in culture medium: 100%, 50%, 25%, 5%, 1% and 0.1%. As a negative control for cytotoxicity, cells were incubated in DMEM culture medium, and as a positive control, 1% Triton-X100 was used. In all cases, 10,000 cells were subcultured in each well of a 96-well plate. Once the cells attached to the culture surface 24 h later, the culture medium was removed and 100 μL of each PGA dilution was added to each well. Therefore, 0.01 μl of each dilution were used per cultured cell. Then all cell viability assays were carried out after 5 different incubation times: 5 and 30 min and 1, 6 and 24 h. All experiments were done in triplicate (3 different cell cultures from 3 donors).

### Determination of cell viability and functional status with WST-1 assays

Cell viability and functional status were determined with the Water-Soluble Tetrazolium Salt-1 (WST-1) colorimetric assay on conjunctival cells incubated on different PGA dilutions for different times (Cell Proliferation Reagent WST-1, Roche Diagnostics, Indianapolis, IN). WST-1 is a tetrazolium dye containing an electron coupling reagent that is cleaved by the mitochondrial dehydrogenase enzyme to a formazan dye. Therefore, the amount of formazan dye formed correlates directly with the number of metabolically active cells in the culture. For this analysis, each well containing the cells was washed twice in PBS, and then 100 μL of DMEM and 10 μL of the cell proliferation reagent WST-1 were added to each well. This mixture was incubated for 4 h at 37°C in a cell culture incubator. Then the plates were agitated for 1 min and absorbance at 450 nm was quantified in a spectrophotometer.

### Determination of cell viability and functional status with MTT assays

To determine cell viability and function with MTT, each well was washed twice in PBS. Then 100 μL DMEM and 10 μL MTT labeling agent were added to each well. This mixture was incubated for 4 h at 37°C in a cell culture incubator. Then 100 μL solubilization solution was added per well and the plates were incubated overnight to allow formazan crystals to solubilize. Finally, absorbance was quantified at 550 nm in a spectrophotometer. This colorimetric nonradioactive assay is based on the metabolic bromide reduction of 3-(4,5-dimethylthiazol-2-yl)-2,5-diphenyl tetrazol (MTT) by functional cells, and is used to determine mitochondrial cell function.

### Determination of cell viability and functional status with a dual cytoplasmic and nuclear calcein/AM-ethidium homodimer-1 assay (LIVE/DEAD)

To simultaneously analyze cell function based on cytoplasmic metabolism and cell viability based on cell membrane integrity, we used the calcein/AM-ethidium homodimer-1 Viability/Cytotoxicity assay kit (LIVE/DEAD, Life Technologies, Carlsbad, CA). For this assay, 20,000 cells were cultured on chamber slides (Lab-Tek Chamber Slides, Nunc, Roskilde, Denmark) and allowed to attach to the slides for 24 h. Then the culture medium was removed and 500 μL of each PGA dilution was added to each chamber. After incubation times of 5 and 30 min, 1, 6 and 24 h, the supernatant was removed and the cells were washed twice with PBS and stained with the LIVE/DEAD assay reagents (calcein/AM and ethidium homodimer-1) (Invitrogen, Carlsbad, CA) according to protocols supplied by Invitrogen. Finally, the number of live (green) and dead (red) cells was determined with a fluorescence microscope, and the percentage of live and dead cells was calculated for each study group. For each culture condition, three determinations were done.

### Statistical analysis

First the dose of each PGA able to reduce cell viability or cell function by 50% was calculated for each compound and each culture period, and an average value was obtained for each PGA (average lethal dose 50, LD50).

Pair-wise comparisons between two study groups (two different PGAs for each incubation time) were done with the Wilcoxon nonparametric test to evaluate the statistical significance of differences between groups. The Kendall tau correlation test was used to establish correlations between variables (times, concentrations and cell viability as determined by the different methods).

A p value of 0.05 was considered statistically significant, and all tests were two-tailed. The SPSS v. 16.00 application was used for all statistical analysis.

## Results

### Cytotoxic effects of PGAs determined by WST-1 assays

**Fig 1:**
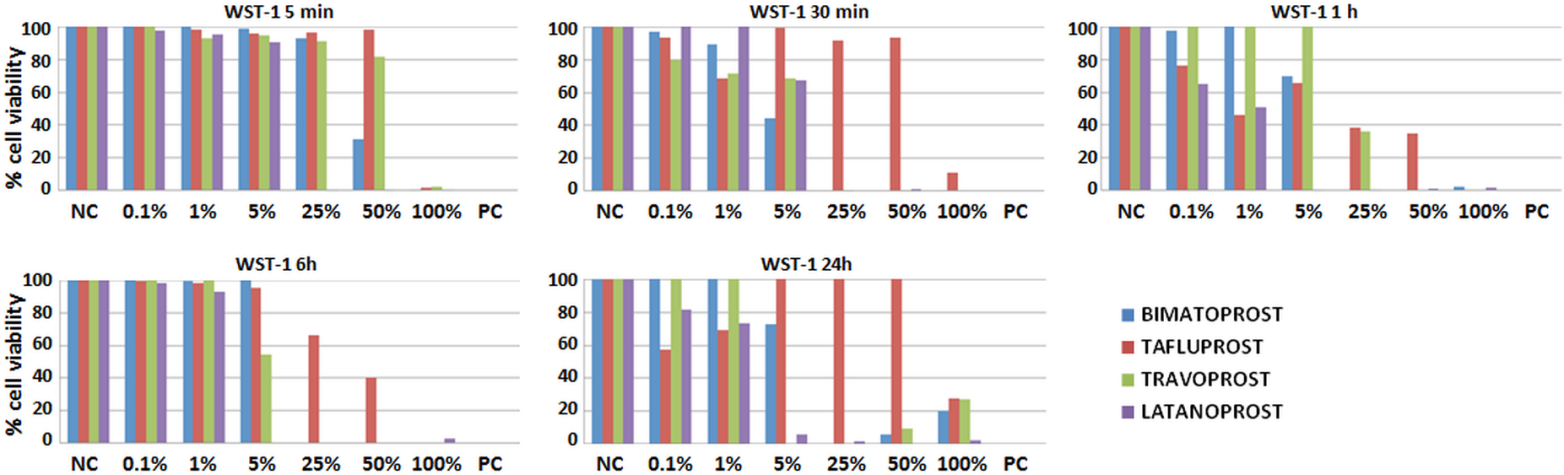
Average cell viability and functional status according to the WST-1 assay in conjunctival cells incubated with different PGA dilutions for different times. NC: negative controls for cytotoxicity; PC: positive controls for cytotoxicity.

Our analysis of cytotoxic cell damage induced by the four PGAs showed that all eye drop formulations had some degree of cytotoxicity. When we compared the metabolic activity of cells incubated with the different drugs to negative controls without PGA using the WST-1 method, we found a statistically significant decrease in cell activity and viability at all incubation times (5 and 30 min, 1, 6 and 24 h) (Table 1 and Fig 1). However, comparisons with positive controls showed that the cytotoxic effects of 0.03% bimatoprost and latanoprost did not differ from those induced by Triton X-100, which was toxic to cells at all incubation times. Similarly, travoprost was highly toxic to conjunctival cells after incubation for 30 min to 24 h, but was significantly less toxic than the positive control at 5 min. In contrast, tafluprost was more toxic that the control (drug-free) culture medium but significantly less cytotoxic than the control Triton X-100. As shown in Table 2, statistical analysis revealed that the effect of all four PGAs was different, although some similarities were found between specific drugs, especially latanoprost and tafluprost. A statistically significant negative correlation was found between cell viability as determined by WST-1 assays and incubation time (p = 0.001, r = −0.222), drug concentration (p = 0.000, r = −0.470) and the specific PGA used (p = 0.028, r = −0.150). Interestingly, our WST-1 results correlated positively with those obtained with the MTT assay (p = 0.000, r = 0.551) and the LIVE/DEAD assay (p = 0.000, r = 0.512).

**Table 1:** Statistical p values (Wilcoxon nonparametric test) for comparisons of the results with the WST-1 assay.

WST-1		Bimatoprost	Tafluprost	Travoprost	Latanoprost	Positive control	Negative control
5 min	Bimatoprost	-	0.120	0.604	0.000*	0.002*	0.033*
	Tafluprost	0.120	-	0.002*	0.000*	0.006*	0.006*
	Travoprost	0.604	0.002*	-	0.000*	0.002*	0.004*
	Latanoprost	0.000*	0.000*	0.000*	-	0.002*	0.937
30 min	Bimatoprost	-	0.000*	0.460	0.000*	0.000*	0.219
	Tafluprost	0.000*	-	0.000*	0.037*	0.006*	0.000*
	Travoprost	0.460	0.000*	-	0.002*	0.000*	0.061
	Latanoprost	0.000*	0.037*	0.002*	-	0.000*	0.031*
1 h	Bimatoprost	-	0.683	0.074	0.003*	0.000*	0.535
	Tafluprost	0.683	-	0.029*	0.000*	0.000*	0.000*
	Travoprost	0.074	0.029*	-	0.000*	0.000*	0.623
	Latanoprost	0.003*	0.000*	0.000*	-	0.000*	0.134
6 h	Bimatoprost	-	0.306	0.179	0.737	0.002*	0.002*
	Tafluprost	0.306	-	0.001*	0.000*	0.002*	0.002*
	Travoprost	0.179	0.001*	-	0.900	0.002*	0.002*
	Latanoprost	0.737	0.000*	0.900	-	0.002*	0.388
24 h	Bimatoprost	-	0.318	0.116	0.000*	0.000*	0.157
	Tafluprost	0.318	-	0.012*	0.001*	0.000*	0.003*
	Travoprost	0.116	0.012*	-	0.000*	0.000*	0.016*
	Latanoprost	0.000*	0.001*	0.000*	-	0.000*	0.760

* Statistically significant p values

**Table 2:** Average lethal dose 50 (LD50) for each prostaglandin analog and each assay.

	WST-1	MTT	LIVE/DEAD
Bimatoprost	16.98%	18.36%	32.56%
Tafluprost	36.77%	51.45%	77.06%
Travoprost	17.15%	14.52%	32.08%
Latanoprost	8.08%	8.52%	31.36%

Calculation of the average LD50 showed that the most cytotoxic drug was latanoprost, which decreased cell viability and metabolism to 50% of the control value at a concentration of just 8.08% of the commercial product. In contrast, the least cytotoxic PGA was tafluprost, which reduced cell activity and viability by 50% at an average concentration of 36.77% (Table 2).

### Cytotoxic effects of PGAs determined by MTT assays

**Fig 2:**
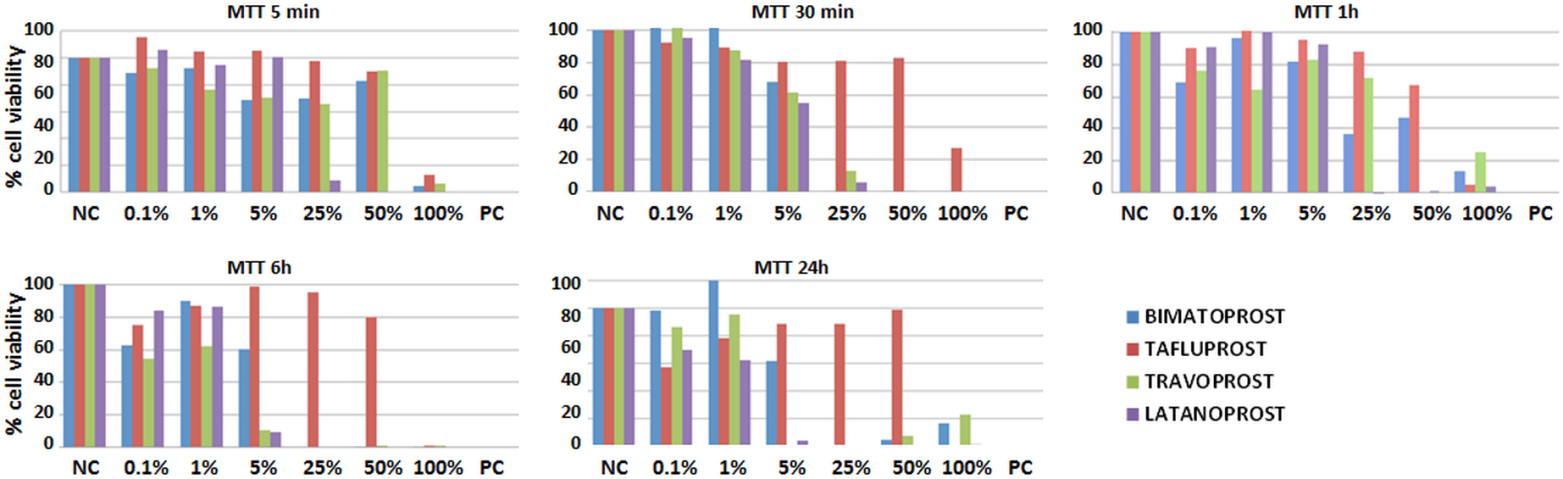
Average cell viability and functional status according to the MTT assay in conjunctival cells incubated with different PGA dilutions for different times. NC: negative controls for cytotoxicity; PC: positive controls for cytotoxicity.

The MTT assay showed that most PGAs reduced cell mitochondrial activity after all incubation periods except for bimatoprost (at 5 and 60 min) and tafluprost (at 5 min and 24 h) (Table 3 and Fig 2). Comparisons of the cytotoxic effects of all four PGAs to the positive control for mortality showed that treatment with bimatoprost, travoprost and latanoprost reduced cell metabolic activity to levels similar to those of the Triton X-100 control at 30 and 60 min, 6 h and 24 h, suggesting that these drugs were as cytotoxic as the control after these periods. However, cells treated with tafluprost for 5, 30 and 60 min, 6 h and 24 h and with bimatoprost and latanoprost for 5 min showed significantly different MTT activity levels compared to the positive control. In addition, statistical comparisons of the four PGAs showed that tafluprost was significantly different from the other three PGAs after most incubation periods. A statistically significant negative correlation was found between cell viability as determined by MTT assays and PGA concentration (p = 0.000, r = −0.485), and between viability and the specific type of PGA (p = 0.001, r = −0.219), but no such correlation was found between viability and incubation time. The results for the MTT assay correlated positively with those for the WST-1 assay (p = 0.000, r = 0.551) and the LIVE/DEAD assay (p = 0.000, r = 0.353).

**Table 3:** Statistical p values (Wilcoxon nonparametric test) for comparisons of the results with the MTT assay.

MTT		Bimatoprost	Tafluprost	Travoprost	Latanoprost	Positive control	Negative control
5 min	Bimatoprost	-	0.000*	0.573	0.209	0.065	0.003*
	Tafluprost	0.000*	-	0.000*	0.000*	0.117	0.003*
	Travoprost	0.573	0.000*	-	0.322	0.002*	0.013*
	Latanoprost	0.209	0.000*	0.322	-	0.003*	0.003*
30 min	Bimatoprost	-	0.003*	0.068	0.001*	0.592	0.002*
	Tafluprost	0.003*	-	0.000*	0.000*	0.002*	0.002*
	Travoprost	0.068	0.000*	-	0.001*	0.637	0.002*
	Latanoprost	0.000*	0.000*	0.001*	-	0.454	0.002*
1 h	Bimatoprost	-	0.000*	0.931	0.068	0.001*	0.002*
	Tafluprost	0.000*	-	0.000*	0.008*	0.180	0.000*
	Travoprost	0.931	0.000*	-	0.198	0.002*	0.003*
	Latanoprost	0.068	0.008*	0.198	-	0.000*	0.486
6 h	Bimatoprost	-	0.000*	0.001*	0.122	0.001*	0.001*
	Tafluprost	0.000*	-	0.000*	0.000*	0.975	0.005*
	Travoprost	0.001*	0.000*	-	0.033*	0.001*	0.001*
	Latanoprost	0.122	0.000*	0.033*	-	0.001*	0.002*
24 h	Bimatoprost	-	0.206	0.116	0.000*	0.000*	0.157
	Tafluprost	0.206	-	0.011*	0.000*	0.011*	0.003*
	Travoprost	0.116	0.011*	-	0.000*	0.000*	0.016*
	Latanoprost	0.000*	0.000*	0.000*	-	0.000*	0.760

* Statistically significant p values

The average LD50 with the MTT method indicated that the most cytotoxic drug was latanoprost, which decreased cell viability and metabolism to 50% of the control value at a concentration of 8.52% of the commercial product. The least cytotoxic PGA was tafluprost, which reduced cell activity and viability by 50% at an average concentration of 51.45% (Table 2).

### Cytotoxic effects of PGAs determined by LIVE/DEAD assays

**Fig 3:**
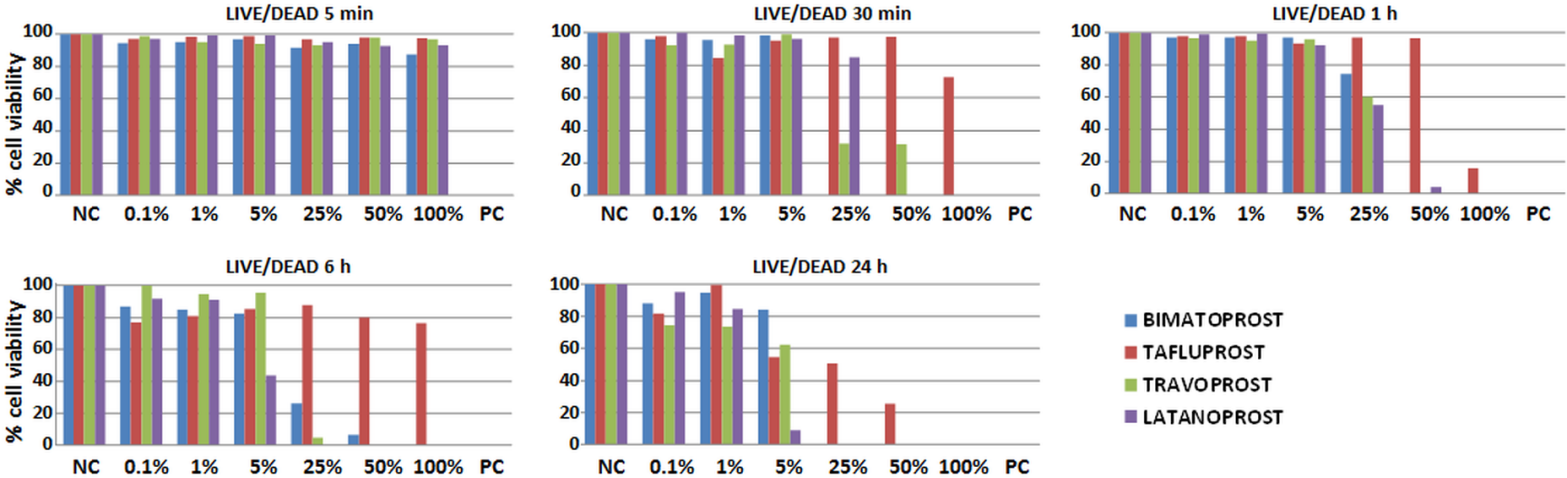
Average cell viability and functional status according to the LIVE/DEAD assay in conjunctival cells incubated with different PGA dilutions for different times. NC: negative controls for cytotoxicity; PC: positive controls for cytotoxicity.

**Fig 4:**
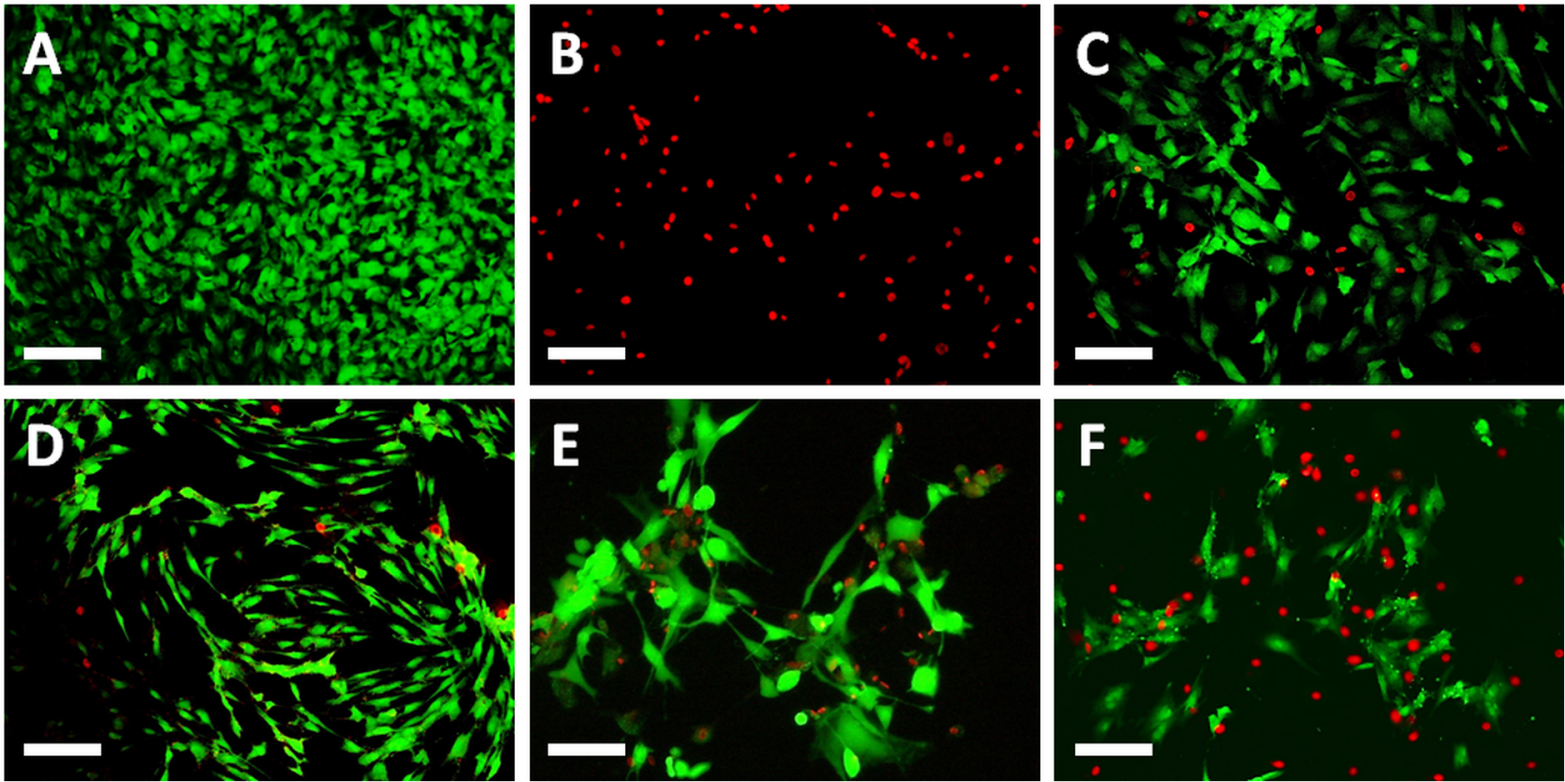
Illustrative images corresponding to the analysis of cell viability and functional status according to the LIVE/DEAD assay in conjunctival cells incubated with different PGAs dilution for different times. A: negative controls for cytotoxicity; B: positive controls for cytotoxicity; C: cells incubated with a 25% concentration of bimatoprost for 1 h; D: cells incubated with a 25% concentration of tafluprost for 1 h; E: cells incubated with a 25% concentration of travoprost for 1 h; F: cells incubated with a 25% concentration of latanoprost for 1 h. Scale bars: 100 μm.

When we analyzed cell viability with the dual cytoplasmic and nuclear cell viability and cell function assay LIVE/DEAD, we found that the results for the PGAs differed significantly from the positive and negative controls, suggesting that all PGAs altered cell viability, albeit to a lesser extent than the Triton X-100 control after all periods (Table 4 and Figs 3 and 4). Our comparison of the cytotoxic effects of the four PGAs showed that tafluprost tended to result in the highest levels of cell viability and function, especially at the highest doses, although the differences between incubation periods were not statistically significant. Statistically significant negative correlations were found between cell viability determined by LIVE/DEAD and incubation time (p = 0.000, r = −0.366), and between viability and the concentration of PGA (p = 0.000, r = −0.451), although no such correlation was found between viability and the type of PGA used. These results correlated positively with those obtained with the WST-1 assay (p = 0.000, r = 0.512) and the MTT assay (p = 0.000, r = 0.353).

**Table 4:** Statistical p values (Wilcoxon nonparametric test) for comparisons of the results with the LIVE/DEAD assay.

LIVE/DEAD		Bimatoprost	Tafluprost	Travoprost	Latanoprost	Positive control	Negative control
5 min	Bimatoprost	-	0.002*	0.028*	0.005*	0.002*	0.002*
	Tafluprost	0.002*	—	0.028*	0.099	0.002*	0.002*
	Travoprost	0.028*	0.028*	-	0.875	0.002*	0.002*
	Latanoprost	0.005*	0.099	0.875	-	0.002*	0.002*
30 min	Bimatoprost	-	0.099	0.331	0.035*	0.002*	0.026*
	Tafluprost	0.099	-	0.049*	0.271	0.002*	0.002*
	Travoprost	0.331	0.049*	-	0.331	0.002*	0.005*
	Latanoprost	0.035*	0.271	0.331	-	0.005*	0.011*
1 h	Bimatoprost	-	0.028*	0.011*	0.506	0.002*	0.011*
	Tafluprost	0.028*	-	0.012*	0.049*	0.002*	0.002*
	Travoprost	0.011*	0.012*	-	0.878	0.002*	0.011*
	Latanoprost	0.506	0.049*	0.878	-	0.002*	0.005*
6 h	Bimatoprost	-	0.099	0.574	0.074	0.002*	0.005*
	Tafluprost	0.099	-	0.157	0.023*	0.002*	0.002*
	Travoprost	0.574	0.157	-	0.011*	0.005*	0.011*
	Latanoprost	0.074	0.023*	0.011*	-	0.002*	0.026*
24 h	Bimatoprost	-	0.574	0.026*	0.113	0.002*	0.026*
	Tafluprost	0.574	-	0.021*	0.012*	0.002*	0.005*
	Travoprost	0.026*	0.021*	-	0.916	0.002*	0.026*
	Latanoprost	0.113	0.012*	0.916	-	0.002*	0.026*

* Statistically significant p values

The average LD50 for the LIVE/DEAD assay confirmed that the PGA formulation with the weakest cytotoxic effect was tafluprost (a 77.06% concentration led to 50% cell death), whereas the average LD50 for the rest of the cytotoxic agents ranged between 31.36% and 32.56% (Table 2).

## Discussion

Glaucoma is a chronic disease that often requires years or decades of medical treatment. For this reason, the effects of drugs used on the ocular surface must be determined in normal human cells and with an array of methods and techniques, and additional studies to compare the four PGAs currently approved for clinical use are needed. Whereas most previous studies used immortalized cell lines to study cell viability [22,28], we used primary cultures of human conjunctival cells isolated from normal donors in material obtained during routine ophthalmic surgical procedures. Normal cells represent a much more physiological system that yields results which can be more directly extrapolated to the clinical situation than those obtained with immortalized cell lines, which usually have important genetic abnormalities [29].

Strikingly, our results revealed that all PGAs were cytotoxic to cultured conjunctival cells, although we found significant differences among the four PGAs analyzed here. As expected, our results showed that both the concentration and the incubation period had crucial effects on cell viability, and cells incubated with high concentrations of PGA for longer periods showed the greatest cell damage. Interestingly, the cytotoxic effect of concentration was more important that incubation time, with a clearer negative correlation in all three methods used here. These results are in agreement with previous reports suggesting that concentration had a greater effect than incubation time in several cell types incubated with different drugs [24,30]. Together, the results indicate a need to design novel PGAs with high effectiveness but free from the cytotoxic effects that we found, or at least to obtain drugs that are functional at low dosages. Care should be taken before extrapolating the results to in vivo situations, since the concentration of each drug and the volume per cell used in the present work could be different to real in vivo environment.

Regarding incubation time, several studies reported that the half-life of this type of eye drops is very short [31,32]. We determined the cytotoxic effects of all four PGAs after short periods (5 min, 30 min and 1 h) to reproduce the pharmacokinetics of these compounds. In addition, to better characterize these PGAs, we also analyzed their effects after longer periods (6 h and 24 h). Although these long incubation times may not reproduce the usual clinical situation, it is important to determine the effects of these commonly used drugs after longer periods which mimic the situation most patients face when they need to use these eye drops for long-term treatment.

In general, our results show that the PGA associated with the best cell viability was tafluprost. We note that this is the only one of the four compounds evaluated here that is preservative-free, whereas the other PGAs contain BAK in their formulation. To reproduce the clinical situation more accurately, we always used commercially available products without separating the active components from the preservatives. Our results should therefore be interpreted with due caution. Ophthalmic eye drops commonly contain BAK, a preservative with established cytotoxicity and side effects both *in vivo* and *in vitro*. It is considered a stimulating factor responsible for inflammation [33], and the long-term use of anti-glaucoma medication with BAK has been shown to induce histopathological changes on the ocular surface. These dose-dependent toxic effects were detected with very low concentrations [24]. BAK has also been implicated in conjunctival allergy, dry eye disease and failure of glaucoma surgery [34–36]. These side effects may be partially responsible for the cytotoxicity of three of the PGAs analyzed here, and support the current trend to omit this preservative from commercial eye drop formulations. In fact, some PGAs have been modified to remove BAK from their composition. As alternatives, new preservatives have been developed such as SofZia or Polyquad, which seem to be less harmful to the eye [25,37]. It would be informative to compare the results of our study with PGAs containing these new preservatives. Moreover, some commercial formulations have been introduced on the market without preservatives, e.g. 0.03% bimatoprost and 0.005% latanoprost. Fewer local side effects would be expected with these products, so compliance with treatment may be better, and the patients’ quality of life may be improved [38,39]. Interestingly, the composition of one of the PGAs (bimatoprost) was changed to reduce the amount of active component from 0.03% to 0.01%, and the concentration of BAK was increased from 0.005% to 0.02% in order to increase the effective concentration of PGA able to reach the internal tissues of the eye. The cytotoxic effects of this formulation should be stigated further.

In summary, we evaluated the cytotoxic effects of four major PGAs by using a combination of three different methods. Our results showed a very good correlation among all three methods and point to the need for novel PGA formulations that are more sparing of the ocular surface. If our results are confirmed by further studies, new clinical protocols based on the use of lower dosages of these PGAs should be explored.
